# Case Report: Transjugular liver biopsy-confirmed lymphoma presenting as hemophagocytic lymphohistiocytosis with severe thrombocytopenia: a case series of three patients

**DOI:** 10.3389/fimmu.2026.1884908

**Published:** 2026-07-27

**Authors:** Luocheng Zhang, Yang Dai, Xushu Zhong, Xuefeng Luo, Ting Niu, Ailin Zhao

**Affiliations:** 1 Department of Hematology, Institute of Hematology, and Center for High Altitude Medicine, West China Hospital, Sichuan University, Chengdu, Sichuan, China; 2 West China Medical School, West China Hospital, Sichuan University, Chengdu, China; 3 State Key Laboratory of Biotherapy, Collaborative Innovation Center of Biotherapy, West China Hospital, Sichuan University, Chengdu, Sichuan, China; 4 National Facility for Translational Medicine (Sichuan), West China Hospital, Sichuan University, Chengdu, China; 5 Division of Gastroenterology and Hepatology, West China Hospital, Sichuan University, Chengdu, Sichuan, China

**Keywords:** diffuse large B-cell lymphoma, extranodal lymphoma, hemophagocytic lymphohistiocytosis, thrombocytopenia, transjugular liver biopsy

## Abstract

**Background:**

Hemophagocytic lymphohistiocytosis (HLH) is a life-threatening hyperinflammatory syndrome that can obscure underlying malignancies, particularly aggressive B-cell lymphoma. Severe cytopenias and coagulopathy often preclude conventional biopsy, delaying diagnosis.

**Objectives:**

To assess the diagnostic utility of transjugular liver biopsy (TJLB) in adult patients presenting with HLH, transfusion-refractory thrombocytopenia, and predominant hepatic involvement, and to explore mechanisms of thrombocytopenia.

**Methods:**

Three adult patients with HLH, hepatic lesions, and severe thrombocytopenia unresponsive to platelet transfusions were retrospectively analyzed. Conventional biopsy was contraindicated due to bleeding risk. TJLB was performed for histopathological confirmation, followed by lymphoma-directed therapy. Laboratory, imaging, and histopathological data were reviewed.

**Results:**

TJLB established aggressive B-cell lymphoma in all patients, enabling timely chemophagocytic therapy. Platelet counts normalized within 2–4 weeks (case 1 and 2), and HLH-related hyperinflammation resolved. Thrombocytopenia was multifactorial, reflecting hemophagocytosis, immune-mediated platelet destruction, hepatic sequestration, and cytokine-mediated suppression of megakaryopoiesis.

**Conclusions:**

HLH may be the first manifestation of occult lymphoma. TJLB is a safe, effective diagnostic strategy in patients with hepatic involvement and high bleeding risk, facilitating early curative therapy. These cases highlight the value of a multidisciplinary, risk-adapted diagnostic approach and provide mechanistic insight into cytopenia in HLH-associated lymphoma.

## Introduction

Hemophagocytic lymphohistiocytosis (HLH) is a rare, life-threatening hyperinflammatory syndrome characterized by uncontrolled immune activation, hypercytokinemia, and multi-organ dysfunction (1). The condition encompasses both primary (familial/genetic) forms and secondary (acquired) forms, with the latter predominantly occurring in adults (2, 3). Secondary HLH is frequently triggered by infections, autoimmune diseases, or malignancies, among which lymphoma represents one of the most important underlying etiologies in adult patients (4). Aggressive B-cell lymphoma, particularly, is a well-documented trigger of HLH and often portends a poor prognosis without prompt initiation of curative-directed therapy.

Timely identification of the underlying cause is essential for guiding appropriate treatment; however, this is frequently precluded by the patient’s critical condition. HLH patients typically exhibit profound cytopenias and coagulopathy, establishing a bleeding diathesis that contraindicates invasive procedures. In those without superficial lymphadenopathy—where hepatic involvement predominates and percutaneous liver biopsy would be the default diagnostic approach—this hemorrhagic risk leaves tissue diagnosis unattainable. The underlying malignancy may therefore remain occult until the disease advances beyond the window for effective intervention, underscoring the urgent need for alternative diagnostic strategies in this vulnerable population.

We herein report a case series of three adult patients with HLH and hepatic lesions presenting with severe, transfusion-refractory thrombocytopenia in whom transjugular liver biopsy—following failure of conventional diagnostic approaches—provided definitive diagnosis of underlying aggressive B-cell lymphoma, thereby enabling transition to curative-directed therapy.

## Case 1

A 36-year-old male patient presented on September 11, 2025, with a 2-month history of fever, cough, and productive sputum. Initial evaluation at a local hospital identified pancytopenia, coagulopathy, and a hepatic lesion interpreted as a liver abscess. Empiric therapy with clindamycin, moxifloxacin, and eltrombopag yielded no improvement. The patient was then transferred, and laboratory evaluation revealed: reticulocyte count 0.2148 × 10¹²/L, hemoglobin 89 g/L, platelet count 29 × 10^9^/L, WBC 3.47 × 10^9^/L, neutrophils 79.1%; TB 30.1 μmol/L, DB 11.9 μmol/L, triglycerides 5.1 mmol/L, LDH 434 U/L; markedly elevated sCD25 18,830 U/mL, ferritin 1,482 ng/mL; IL-10 3,395.59 pg/mL, IL-6 73.4 pg/mL, with other cytokines relatively normal. AFP 1.74 ng/mL, DCP 24.00 mAU/mL. Imaging revealed multiple hepatic masses: a dominant 10.3 × 6.8 × 12.0 cm lesion in the right posterior lobe and a 3.9 × 3.5 × 3.0 cm lesion in the right anterior lobe, with slight splenomegaly. PET/CT showed intense FDG uptake in the liver and portocaval lymph nodes. Bone marrow examination was unremarkable.

The patient met five of eight HLH-2004 criteria (**Table 1**). ED regimen (etoposide 100 mg twice weekly, dexamethasone 10 mg daily) was initiated. Imaging and labs suggested lymphoma rather than hepatocellular carcinoma. Hepatic lesions were the sole measurable site, but thrombocytopenia remained refractory to ED therapy, eltrombopag, and transfusions, precluding percutaneous biopsy.

**Table 1:** HLH diagnostic criteria fulfilled by three cases.

HLH-2004 diagnostic criteria		Case 1	Case 2	Case 3
Fever (>38.5 °C)		Yes (Maximum 38.5 °C, >1 week)	Yes (Maximum 39.8 °C; >1 week)	Yes (Maximum 38.7 °C; >1 week)
Splenomegaly		Yes (on abdominal CT)	Yes (on abdominal CT)	Yes (on abdominal CT)
Cytopenias (Affecting >=2 lineages)	Hemoglobin (<90 g/L)	No (89 g/L)	Yes (74g/L)	Yes (77 g/L)
	Platelet (<100 × 10^9/L)	Yes (29×10^9/L)	Yes (54×10^9/L)	Yes (49×10^9/L)
	Neutrophils (<1.0 × 10^9/L)	No	No	No
Hypertriglyceridemia (Triglycerides >=3.0 mmol/L or >=265 mg/dL)		Yes (Triglycerides 5.1 mmol/L)	No	No
or Hypofibrinogenemia (Fibrinogen <=1.5 g/L)		No	No	Yes (Fibrinogen 1.32 g/L)
Ferritin (>=500 ng/mL)		Yes (1,482 ng/mL)	Yes (1,160 ng/mL)	Yes (12,459 ng/mL)
sCD25 (>=2,400 U/mL)		Yes (18,830 U/mL)	Yes (3,533 U/mL)	Yes (15,838 U/mL)
Hemophagocytosis (bone marrow, spleen, or lymph nodes)		No	Yes (bone marrow)	No
Decreased or absent natural killer (NK) cell activity		NA	NA	NA

Following multidisciplinary discussion, transjugular liver biopsy (TJLB) was performed on October 17, 2025, via the right internal jugular vein using the Seldinger technique. On the day of the procedure, the platelet count was 15 × 10^9^/L. Multiple core samples were obtained under fluoroscopic guidance; the introducer sheath was retained temporarily. The procedure was successful and well tolerated, with no complications. The sheath was removed on post-operative day three without bleeding.

Histopathology revealed medium-to-large atypical lymphoid cells: CD20+, CD19+, CD22+, CD3-, CD5-, CD30-, CD10-, BCL-6+, MUM-1+, BCL-2+ (>90%), c-MYC+ (10–20%), Ki-67+ (80–85%), EBER negative. Clonal IGH and IGK rearrangements confirmed non-GCB DLBCL; FISH showed no c-MYC, BCL-2, or BCL-6 rearrangements. NGS revealed no actionable variants. ED therapy was discontinued, and R-CHOP was initiated (rituximab 800 mg day 1, cyclophosphamide 1,400 mg day 2, epirubicin 120 mg day 2, vindesine 4 mg day 2, prednisone 50 mg twice daily day 2–6). Platelets recovered to 142 × 10^9^/L within two weeks. Four cycles of R-CHOP achieved complete metabolic response (Deauville 1) on PET/CT, with no HLH relapse. The patient remains on ongoing chemophagocytic therapy (**Figure 1**).

**Figure 1:**
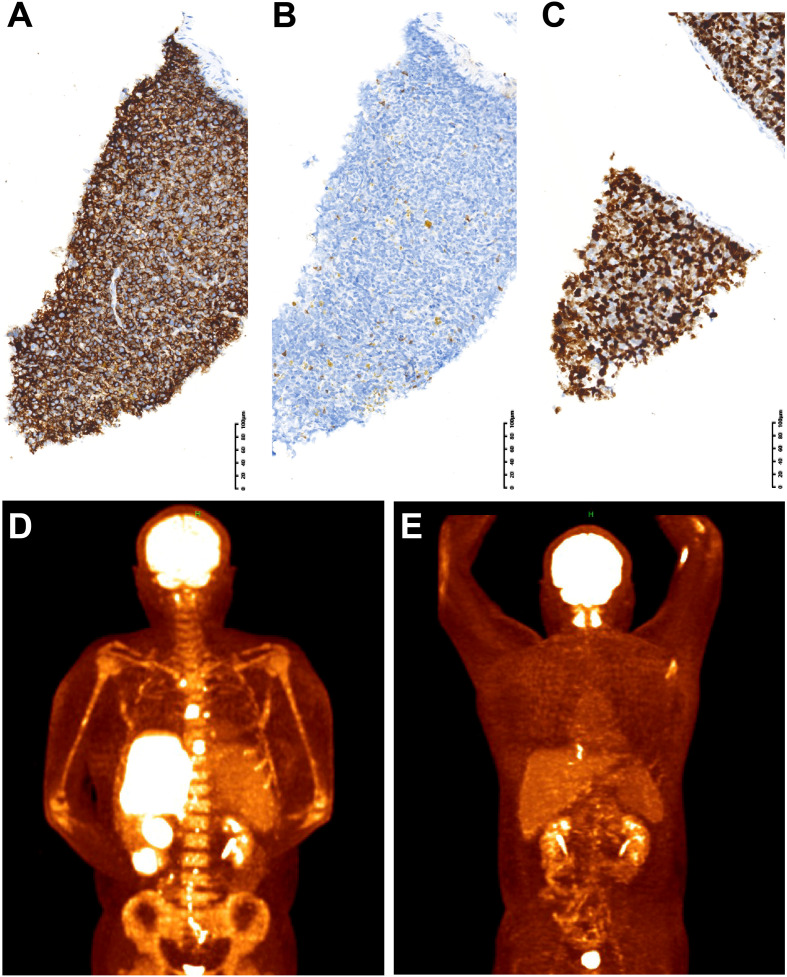
Pathological and PET/CT images of case 1. **(A–C)** Immunohistochemophagocyticical staining of liver biopsy specimens (original magnification ×20): **(A)** CD20 showing diffuse strong positivity in tumor cells; **(B)** CD3 negative in tumor cells; **(C)** Ki-67 demonstrating a high proliferation index. **(D)** Baseline whole-body ^18^F-FDG PET/CT showing intense hypermetabolic activity in the liver and lymph nodes. **(E)** Post-treatment ^18^F-FDG PET/CT demonstrating a marked reduction in metabolic activity.

## Case 2

A 60-year-old male patient presented on December 17, 2025 with a 3-month history of recurrent fever (maximal 39.8 °C) and recent-onset melena. Initial laboratory studies at an outside institution revealed: hemoglobin 78 g/L, platelets 52 × 10^9^/L, WBC 9.19 × 10^9^/L, alkaline phosphatase 171 U/L. Contrast-enhanced CT of the abdomen demonstrated normal hepatic morphology with homogeneous parenchymal attenuation; a hypodense mass measuring approximately 10.2 × 7.4 cm in the left hepatic lobe with confluent appearance; and a hypodense nodule measuring approximately 1.8 × 1.4 cm in the caudate lobe. Mild splenomegaly was also noted. Surgical intervention had originally been planned but was deferred due to persistent fever. Whole-body PET/CT was then performed and demonstrated abnormal FDG uptake in the anterior wall of the left maxillary sinus, liver, ascending colon, small intestine, and bilateral cervical/axillary/abdominal lymph nodes, highly suggestive of hemophagocytic atologic malignancy. On December 4, 2025, an ultrasound-guided axillary lymph node biopsy was attempted but failed to obtain diagnostic tissue.

The patient was subsequently transferred to our hospital. Laboratory studies revealed a hemoglobin level of 78 g/L, platelet count of 52 × 10^9^/L, and white blood cell count of 9.19 × 10^9^/L, with neutrophils of 80%. Biochemophagocyticistry showed elevated alkaline phosphatase at 171 U/L. The coagulation profile was normal. Ferritin 1,374 ng/mL, sCD25 3,533 U/mL; IL-10 1514.48pg/mL, IL-6 48.40pg/mL, while the rest of the cytokines were normal; Tumor markers (AFP, CEA, NSE, SCC, CYFRA21-1) were all within normal limits.

This patient presented with persistent fever, splenomegaly, bicytopenia (hemoglobin 74 g/L, platelets 54 × 10^9^/L) as well as markedly elevated ferritin and sCD25, which prompted the diagnosis of HLH (**Table 1**). The ED regimen (etoposide 100 mg twice weekly, dexamethasone 10 mg daily) was initiated. Concurrently, gastrointestinal bleeding required fasting, acid suppression, somatostatin, and repeated platelet transfusions. Additionally, romiplostim and thrombopoietin receptor agonists were administered to improve the platelet count. Similar to the preceding case, this patient presented with HLH and hepatic lesions, with lymphoma strongly suspected as the underlying etiology and severe thrombocytopenia refractory to platelet transfusion and multiple treatments. However, in contrast to Case 1, the dominant mass was located in the left hepatic lobe, rendering transjugular liver biopsy (TJLB), typically optimized for right lobe sampling, technically more challenging. This anatomical consideration created an additional diagnostic challenge.

Following extensive multidisciplinary discussion involving interventional gastroenterology, TJLB was ultimately attempted with external ultrasound guidance to direct the biopsy needle toward the left hepatic lesion, thereby maximizing the probability of obtaining diagnostic tissue from the pathological site. On December 30, 2025, a TJLB was performed using the same technique as previously described. The biopsy needle was advanced through the left hepatic vein wall into the liver parenchyma to obtain multiple tissue samples. A transabdominal ultrasound was used to confirm the position of the needle tip within the mass, and hemostasis was achieved with manual compression at the puncture site. The procedure was well tolerated, with no hemorrhagic complications.

Histopathological analysis of the liver biopsy specimen established the diagnosis of diffuse large B-cell lymphoma, non-germinal center B-cell-like subtype, with BCL-2 and c-MYC double expression, according to the Hans algorithm. Immunohistochemophagocyticistry demonstrated CD20+, CD79a+, CD3-, CD10-, BCL-6+, MUM-1+, cyclin-D1-, TDT-, PCK-, CD34-, S100-, CD23-, CD30-, CD5-, BCL-2+ (>80%), c-MYC+ (~40%), Ki-67+ (70–80%), with clonal IGH and IGK rearrangements. EBER was negative. FISH analysis yielded no BCL-2, BCL-6, or c-MYC rearrangements. Once the diagnosis was established, the ED regimen was discontinued and CR-CHOP (chidamide 20mg twice weekly, rituximab 600mg day 1, cyclophosphamide 1000mg day 2, epirubicin 80mg day 2, vindesine 4mg day 2, prednisone 50mg bid day2-6) was initiated on January 20, 2026 (5). The patient was discharged in stable condition following platelet recovery to 86 × 10^9^/L, with resolution of melena and afebrile status. The patient is currently receiving ongoing chemotherapy and has not yet reached the mid-treatment evaluation. Platelet counts remained within the normal range during the inter-treatment intervals (**Figure 2**).

**Figure 2:**
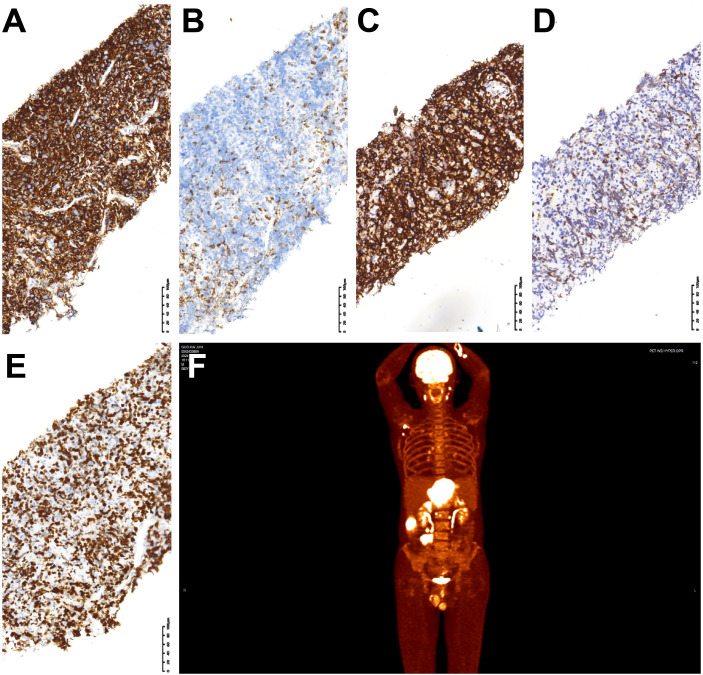
Pathological and baseline PET/CT images of case 2. **(A–E)** Immunohistochemophagocyticical staining of liver biopsy specimens (all images at original magnification ×20): **(A)** CD20 showing diffuse strong positivity in tumor cells; **(B)** CD3 negative in tumor cells; **(C)** BCL-2 demonstrating strong expression (>80%); **(D)** c-MYC showing partial positivity (~40%); **(E)** Ki-67 indicating a high proliferation index. **(F)** Baseline whole-body ^18^F-FDG PET/CT demonstrating intense hypermetabolic activity in the liver and lymph nodes.

## Case 3

A 61-year-old woman presented on March 30, 2026, with a 2-month history of fever, worsening over the past week, accompanied by anorexia, fatigue, abdominal distension, and diarrhea. Initial evaluation revealed ANA 1:320, IL-10 >1000 pg/mL, IL-6–24 pg/mL, TNF-α 12.8 pg/mL, IFN-γ 12.1 IU/mL, ferritin >2000 ng/mL, and sCD25–7086 U/mL. A diagnosis of undifferentiated connective tissue disease was considered and methylprednisolone administered, leading to symptomatic improvement. Fever recurred a week prior to admission, with pallor on examination. Laboratory studies showed hemophagocyticoglobin 77 g/L, WBC 15.19 × 10^9^/L, platelets 21 × 10^9^/L; PT 21.0 s, APTT 54.1 s, fibrinogen 0.9 g/L; TB 68.3 μmol/L, DB 52.9 μmol/L, triglycerides 2.2 mmol/L, LDH 992 U/L, albumin 22.7 g/L, globulin 17.8 g/L, ferritin 12,459 ng/mL, sCD25 15,838 U/mL. Abdominal CT revealed diffuse hepatic changes with hepatosplenomegaly, ascites, and multiple enlarged lymph nodes.

Given persistent fever, splenomegaly, bicytopenia, and markedly elevated ferritin and sCD25, HLH was diagnosed (**Table 1**). ED therapy (etoposide 80 mg twice weekly, dexamethasone 10 mg daily) was initiated. EBV-DNA was undetectable; bone marrow studies were unremarkable. PET/CT showed hepatosplenomegaly with diffuse FDG uptake and small lymph nodes with intense FDG avidity (SUVmax 13.15; **Figure 3**), raising suspicion for lymphoma. Due to high hemophagocytic risk, TJLB was performed. Post-procedure, platelets fell to 2 × 10^9^/L. Histopathology revealed focal atypical cells: CD20+, CD3-, CD5 (partial), Bcl-6+, MUM-1+, Bcl-2+ (majority), c-MYC+ (few), Ki-67 40–50%, EBER1/2 negative, and clonal IGH/IGK rearrangements, consistent with non-GCB DLBCL.

**Figure 3:**
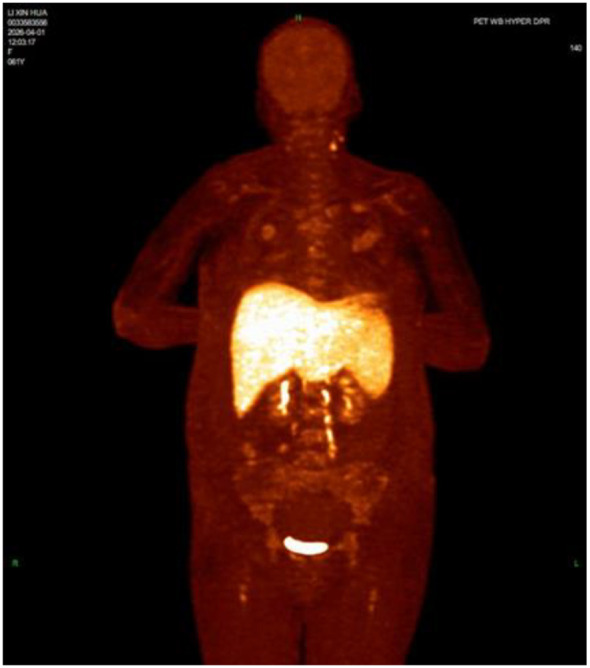
Baseline PET/CT image of case 3. Baseline whole-body ^18^F-FDG PET/CT demonstrating intense hypermetabolic activity in the liver, spleen and lymph nodes.

While awaiting pathology, ED therapy improved fever, ferritin, sCD25, and bilirubin, but cytopenias persisted and progressed to agranulocytosis. Etoposide was discontinued and G-CSF was administered; however, a severe pulmonary infection developed, requiring ICU care. Consequently, lymphoma-directed treatment was temporarily deferred due to infection, and the platelet count remained below normal at the end of the observation period (19 × 10^9^/L on April 23, 2026; **Figure 4**).

**Figure 4:**
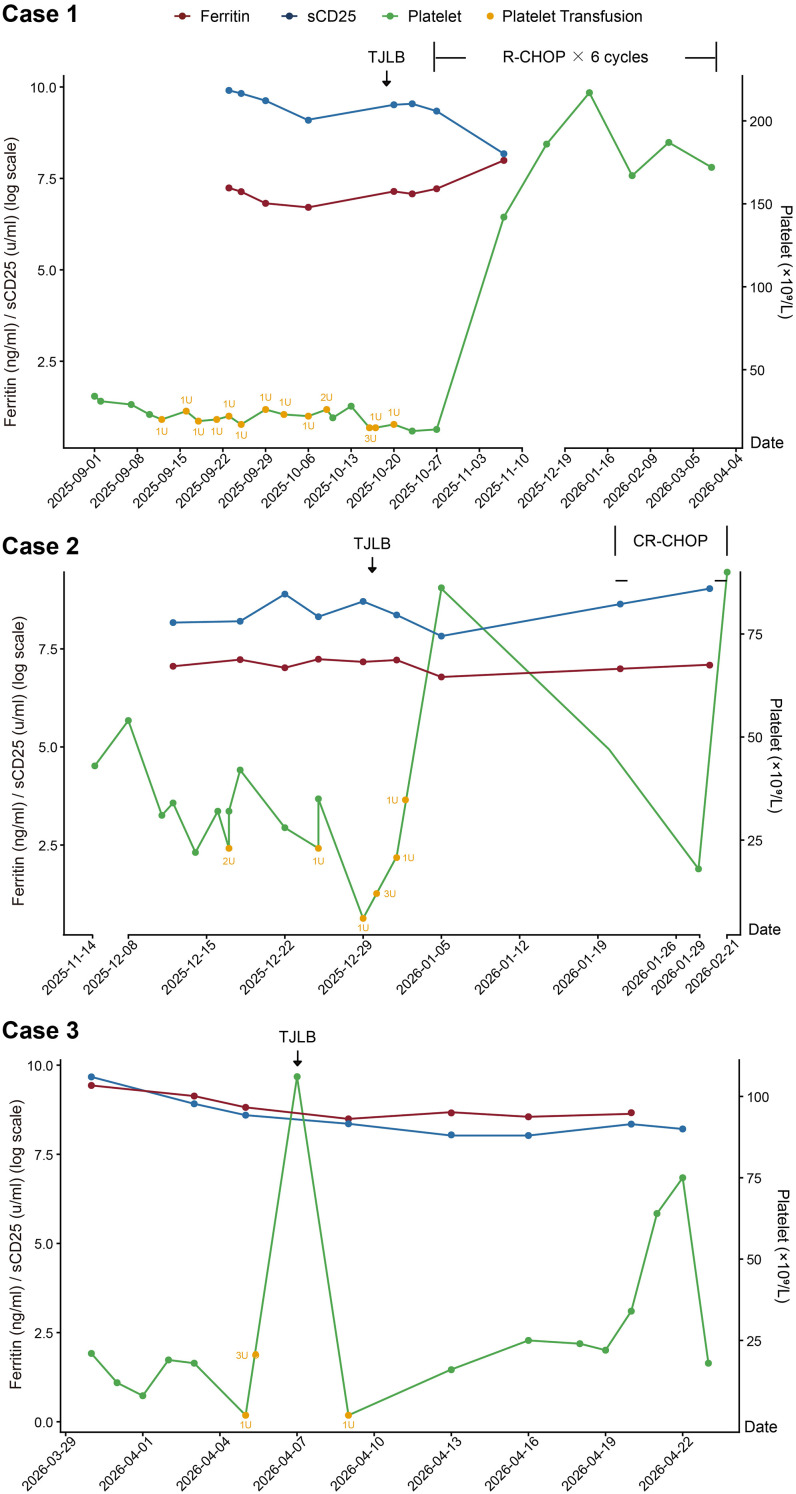
Laboratory parameters following therapy in three cases. Ferritin (red) and sCD25 (blue) levels are shown on a logarithmic scale (left y-axis), while platelet counts (green, right y-axis) are presented over time. Platelet transfusions are indicated by orange dots with corresponding units administered. In all three cases, patients presented with hyperinflammatory features consistent with HLH, including persistently elevated ferritin and sCD25. Despite repeated platelet transfusions, severe thrombocytopenia remained refractory. Transjugular liver biopsy (TJLB) was performed in each case, leading to the definitive diagnosis of underlying lymphoma. Following initiation of lymphoma-directed chemotherapy (Case 1: R-CHOP ×6 cycles; Case 2: CR-CHOP), platelet counts recovered and inflammatory markers gradually declined.

## Discussion

Liver involvement in lymphoma-associated HLH has been previously documented, including a case of primary hepatic lymphoma presenting as HLH and diagnosed by laparoscopic liver biopsy (6). However, in the context of HLH, this procedure remains associated with a substantial risk of bleeding. TJLB is a safe and less invasive alternative to laparoscopic liver biopsy, with proven effectiveness in patients who have bleeding tendencies from various causes, including Fontan-associated liver disease and unexplained liver dysfunction (7, 8). Despite these advantages, its use in lymphoma-associated HLH with predominant hepatic involvement—where conventional percutaneous biopsy is contraindicated—has been rarely described.

Several aspects of these cases merit attention. The profound, transfusion-refractory thrombocytopenia observed exceeds typical HLH coagulopathy, reflecting multifactorial causes including immune-mediated destruction, splenic/hepatic sequestration, and cytokine-driven impaired megakaryopoiesis. Aggressive B-cell lymphomas, especially with extranodal or sinusoidal involvement, can present as a “masked” syndrome dominated by systemic inflammation. The present cases highlight the critical role of TJLB in this high-risk setting, demonstrating that a multidisciplinary, risk-adapted biopsy strategy can achieve timely tissue diagnosis without incurring major hemorrhagic complications (**Figure 4**).

### Mechanisms of thrombocytopenia in hepatic lymphoma-associated HLH

In this setting, thrombocytopenia is likely multifactorial. We hypothesize that thrombocytopenia potentially reflects consumption, immune-mediated destruction, sequestration, and impaired production.

In HLH, activated macrophages engulf platelets and other blood cells, contributing to cytopenias and poor response to transfusion. In B-cell lymphoma, lymphoma cells may produce autoantibodies, further accelerating immune-mediated platelet clearance6. Mild splenomegaly suggested additional sites of platelet sequestration, with the liver playing a key role: lymphoma infiltration and systemic inflammation can cause sinusoidal congestion, Kupffer cell activation, and altered intrahepatic blood flow, promoting platelet trapping. Inflammation-driven endothelial activation may also increase microvascular platelet adhesion and consumption (9). A central feature of HLH is dysregulated cytokine release driven by excessive macrophage and cytotoxic T-cell activation (10). Hypercytokinemia, particularly elevated IL-10 and IL-6, reflects immune dysregulation and may suppress megakaryopoiesis, compounding peripheral platelet loss. These proposed mechanisms could collectively account for the profound, transfusion-refractory thrombocytopenia observed in our cases.

### Pathophysiological convergence of extranodal B-cell lymphoma and HLH

HLH is a well-recognized presentation of aggressive B-cell lymphomas, particularly those with a tendency for extranodal involvement, including hepatic infiltration by diffuse large B-cell lymphoma (11). In this context, HLH-related manifestations frequently dominate the clinical presentation. Systemic hyperinflammation, cytopenias, and organ dysfunction may overshadow the underlying malignancy, making early recognition challenging (10, 12). Key markers such as ferritin and sCD25 are elevated in both conditions, reflecting systemic inflammation and immune activation. Ferritin level frequently exceeds 5000 ng/mL in HLH and may rise comparably in lymphoma-related cases (13). Similarly, sCD25, a surrogate marker of T-cell activation, is commonly increased in both settings. These overlapping biochemophagocytic features complicate the distinction between non-neoplastic HLH and lymphoma-related HLH, thereby reducing clinical suspicion for lymphoma and delaying diagnosis.

The pathophysiological convergence of extranodal B-cell lymphoma and HLH operates through multiple interconnected mechanisms (14). Although the latter two patients in our series presented with lymph node involvement, they also exhibited extranodal disease, predominantly hepatic involvement. Malignant B cells within extranodal microenvironments, particularly the liver, establish sustained crosstalk with the innate immune system through aberrant cytokine secretion (15). Tumor-associated macrophages and malignant B cells themselves produce excessive interleukin-6, interleukin-10, and interferon-γ, which collectively suppress cytotoxic T-cell and natural killer cell function while driving macrophage activation and hemophagocytosis (16). In hepatic involvement, lymphomatous infiltration of the sinusoidal spaces disrupts normal Kupffer cell homeostasis, converting these resident macrophages into hyperactivated effectors that engage in uncontrolled phagocytosis (1). The liver’s unique immunological architecture, with its dense reticuloendothelial network and continuous exposure to gut-derived antigens, renders it particularly susceptible to this malignant cell-macrophage interplay (17). Furthermore, the absence of lymph node filtering in these extranodal presentations—as exemplified by our first patient—allows malignant cells to directly access the systemic circulation, disseminating inflammatory mediators that amplify the hypercytokinemic state across multiple organ systems (**Figure 5**).

**Figure 5:**
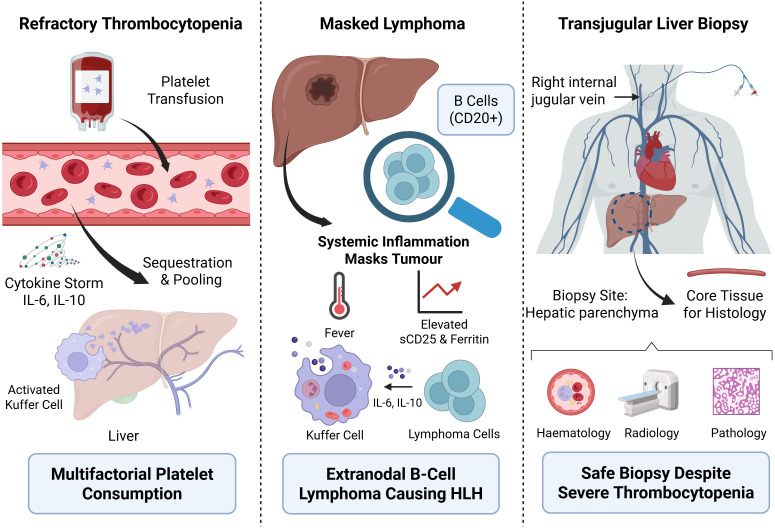
Proposed diagnostic and pathophysiological framework. The schemophagocyticatic illustrates the interplay between hyperinflammation, platelet consumption and occult lymphoma in patients presenting with HLH-like features. Left panel: Refractory thrombocytopenia is driven by multifactorial platelet consumption, including cytokine-mediated activation (e.g., IL-6, IL-10), hepatic sequestration and pooling, and macrophage/Kupffer cell activation. These mechanisms contribute to poor platelet increment despite repeated transfusions. Middle panel: Top:Underlying extranodal B-cell lymphoma (CD20+) is masked by systemic inflammation. Elevated ferritin and sCD25, together with fever and cytokine release, reflect HLH activity and obscure the malignant process. Bottom: Lymphoma cell–derived cytokines (e.g., IL-10, IL-6) stimulate hepatic Kupffer cells, further exacerbating HLH. Right panel: Transjugular liver biopsy via the right internal jugular vein enables safe acquisition of hepatic tissue for histopathological diagnosis, even in the setting of severe thrombocytopenia. Integration of hematological, radiological and pathological assessment allows definitive identification of lymphoma.

### A multidisciplinary, risk-adapted application of TJLB in high-risk patients

In patients with severe thrombocytopenia and coagulopathy, such as those with HLH, conventional percutaneous liver biopsy is often contraindicated due to the substantially increased risk of hemorrhagic complications (18). In this context, TJLB has emerged as a safer alternative, as it avoids capsular puncture and allows tissue acquisition via the hepatic venous system (19). Consistent with this, TJLB is primarily indicated in patients with coagulation disorders, thrombocytopenia, or ascites, where percutaneous approaches are considered high risk (20). From a safety perspective, both systematic reviews and large cohort analyses have demonstrated a favorable risk profile for TJLB. A comprehensive review including over 7,000 procedures reported major complication rates of approximately 0.5%, supporting its safety even in high-risk populations. Similarly, propensity-matched analyses have shown that TJLB is associated with a lower risk of bleeding-related complications, such as hemophagocyticatoma, compared with percutaneous biopsy. In parallel, diagnostic yield remains high, with histological diagnosis achieved in over 95% of cases despite a smaller specimen size (18). Beyond its safety, the successful implementation of TJLB in such high-risk settings relies heavily on coordinated multidisciplinary collaboration. The procedure requires integration of expertise from hematology (risk stratification and peri-procedural management), hepatology (indication and interpretation), and interventional radiology (technical execution under imaging guidance). Pre-procedural imaging plays a critical role in defining vascular anatomy and optimizing puncture trajectory, thereby improving technical success and minimizing complications.

Taken together, TJLB in this context should not be viewed merely as a technical alternative, but rather as part of a multidisciplinary, risk-adapted diagnostic strategy. This approach expands the diagnostic options for critically ill patients with severe bleeding risk, while maintaining both procedural safety and diagnostic reliability. However, it is important to acknowledge that these observations are drawn from a small case series of only three patients, which inherently limits the generalizability of our findings. Caution should be exercised when extrapolating these results to broader populations, and larger prospective studies are needed to further validate the safety and diagnostic utility of TJLB in this specific clinical scenario.

## Conclusion

This case series underscores the formidable diagnostic challenges posed by HLH occurring as the inaugural manifestation of occult aggressive B-cell lymphoma with hepatic involvement. Our findings highlight the multifactorial nature of thrombocytopenia in this context. Moreover, the propensity of extranodal B-cell lymphomas—particularly those with sinusoidal hepatic tropism—to present as “masked” inflammatory syndromes devoid of overt lymphadenopathy necessitates heightened clinical vigilance. The diagnostic trajectory of these patients demonstrates that a multidisciplinary, risk-adapted approach incorporating TJLB can circumvent the prohibitive hemorrhagic risk of percutaneous sampling in the setting of severe bleeding diathesis, yielding definitive histopathological diagnosis and enabling timely initiation of lymphoma-directed therapy.

## Data Availability

The raw data supporting the conclusions of this article will be made available by the authors, without undue reservation.
